# A sequential strategy of upfront radiofrequency ablation followed by endoscopic papillectomy for complex ampullary tumors

**DOI:** 10.3389/fmed.2026.1835891

**Published:** 2026-06-19

**Authors:** Benhua Wu, Lisheng Wang, Wenbiao Chen

**Affiliations:** Department of Gastroenterology, Shenzhen People’s Hospital, The Second Clinical Medical College, Jinan University, Shenzhen, China

**Keywords:** ampullary tumors, endoscopic papillectomy, radiofrequency ablation, sequential application, adverse events

## Abstract

**Background:**

Endoscopic papillectomy (EP) is the first-line treatment for ampullary tumors, and radiofrequency ablation (RFA) as an alternative or adjunctive treatment. This study systematically analyzed EP’s clinical outcomes and evaluated the feasibility and safety of a sequential RFA-first strategy for complex ampullary tumors.

**Methods:**

We retrospectively analyzed 132 patients treated with EP or RFA at Shenzhen People’s Hospital from April 2018 to August 2024. Categorical variables were presented as frequencies (percentages), and continuous variables as mean ± Standard Deviation (SD). Binary logistic regression analyzed risk factors. Propensity score matching and analysis of variance (ANOVA) compared adverse events between treatments.

**Results:**

Initially, 123 of 132 patients underwent EP, while 9 received RFA as the initial treatment due to tumors >3 cm, peri-diverticular lesions, and intraductal invasive lesions. The technical success rate was 100%, and the overall short-term clinical success rate was 96.6%. Adverse events occurred in 20.5% of the patients. Tumor size was identified as a significant risk factor for adverse events such as postprocedural pancreatitis (*p* = 0.03), intraprocedural bleeding (*p* < 0.001), and postprocedural bleeding (*p* = 0.024). The short-term clinical success rate of the RFA group was 91.7%, while adverse events were observed in 16.7% of the patients. Significantly, our findings revealed a statistically no significant difference in adverse events between the RFA and non-RFA groups (*p* = 0.719).

**Conclusion:**

An upfront RFA strategy followed by staged EP or additional RFA may be a potentially feasible and safe method to reduce adverse events in select patients with complex ampullary tumors. However, long-term efficacy and late complication rates require further evaluation with extended follow-up.

## Introduction

Ampullary tumors is a rare and insidious disease in clinic. Because the malignant change rate of this disease is 40–60%, and it could develop from adenoma to adenocarcinoma, therefore, the realization of early complete resection is of great clinical significance. Whereas, ampullary tumor poses great challenges to clinical management due to its unique location (1). Traditionally, these tumors have been managed surgically, primarily through pancreatoduodenectomy (PD), a procedure associated with significant morbidity and mortality. Recently, endoscopic papillectomy (EP) has emerged as a less invasive first-line treatment, offering lower complication and mortality rates (2, 3). However, the practical application of EP in ampullary tumors is challenging. The incidence of adverse events in EP treatment was between 36.6 and 45.4%, especially higher risk associated with larger tumors (2–7). Choi et al. reported that tumors ≥20 mm have a 9.61 times higher bleeding risk (4). The investigation from Lorenzo et al. revealed that recurrence rates after EP treatment range from 14 to 31% (8, 9).

Radiofrequency ablation (RFA) has been investigated as an adjunct to EP, particularly application for residual or recurrent ampullary tumors (10, 11). Previous study found the cure rates (defined as the absence of residual or recurrent tumors at last follow-up) of RFA treatment, range between 70 and 90% (12, 13). Even, RFA complications include postprocedural pancreatitis and stricture formation, its bleeding risks significantly lower than EP’s, possibly due to RFA’s coagulative effect (3, 5, 10, 12, 14–18). Current strategies often involve initial EP followed by RFA for residual or recurrent lesions, typically in a single-session RFA (19, 20). Although the combined therapy of EP and RFA may increase complications, such as pancreatitis. Whereas studies suggest no significant difference in complication rates between combined therapy and EP alone (19).

The size of large ampullary tumor is big challenging for EP treatment due to high risk of post-EP bleeding (4, 21). Initial RFA can reduce the size of tumor, which enabling a subsequent EP performed at least 1 month after RFA, with a minimized bleeding risk. In addition, EP can remove most of the ampullary tumor involving the bile duct. It is noteworthy that RFA supplementation therapy after 1 month could targeting residual intraductal lesions and reduce the risk of pancreatitis. Furthermore, recurrent cases treated with EP could be cured or the probability of recurrence could be reduced through multiple RFA sessions. The EP and RFA method plays an important role in the treatment of ampullary tumors, but there are limited systematic studies on the treatment of EP in ampullary tumors, the strategy of EP and RFA combined therapy, and the comparative study of EP and RFA in the treatment of ampullary tumors.

Here, we systematically analyzed the clinical features, safety and adverse events of EP in the treatment of ampullary tumors. And The efficacy and safety of EP and RFA in the treatment of ampullary tumors were compared. Our findings indicate that the sequential application of EP and RFA could be a potentially safe and effective strategy for managing selected cases of ampullary tumors with complex features.

## Materials and methods

### Study design

This retrospective study was conducted at Hospital and included consecutive patients diagnosed with ampullary tumors who underwent endoscopic treatment between April 2018 and August 2024. Patients with a follow-up duration of <2 months were excluded to ensure the validity and reliability. Data collection included the demographic, clinical, lesion, procedural, and follow-up outcomes. Demographics included age, sex, and comorbidities; clinical data included presenting symptoms and prior interventions. Clinical data encompassed presenting symptoms and prior interventions. Lesion characteristics were based on endoscopic appearance, histology, and intraductal extension. Procedural data covered technical aspects, duration, and immediate complications of EP and RFA. Follow-up outcomes focused on tumor recurrence and further intervention needs. The flow chart of this research is shown in Supplementary Figure S1.

### Definitions

Remnant lesions were defined as adenomatous tissue detected by histology during the initial postprocedural surveillance endoscopy, indicating incomplete resection (8, 9, 16). Recurrent lesions were defined as the reappearance of tumors in follow-up endoscopies after no remnant lesions were detected in the initial surveillance endoscopy (17, 22). Technical success was defined as the successful completion of the intended procedures (EP or RFA) and achievement of therapeutic goals (3). short-term clinical success was defined as complete lesion removal, regardless of the number of sessions, including cases where recurrence was managed with additional treatment, with no histological evidence of residual or recurrent lesions (19). Due to the short follow-up period, the long-term clinical success cannot be predicted at present. Intraprocedural bleeding was defined as bleeding that persisted for >3 min after the EP or RFA procedures, requiring endoscopic intervention to achieve hemostasis (21). Postprocedural bleeding was defined as any of the following >4 h post procedure in patients with initially hemostasis, including any of the following: overt bleeding (hematemesis, melena, hematochezia, or blood in the nasogastric tube aspirate), hemodynamic instability from bleeding, hemoglobin drop >2 g/dL; and endoscopic evidence of active bleeding from the papilla of Vater (2, 12, 15).

### Patient selection criteria for initial RFA

Initial RFA without concurrent EP was considered in patients meeting at least one of the following criteria: (1) tumor size >3 cm, as large lesions are associated with higher bleeding risk and incomplete resection; (2) peri-diverticular location of the ampullary tumor, which increases technical difficulty and risk of perforation during snare resection; (3) evidence of intraductal invasive extension on endoscopic ultrasonography (EUS) or magnetic resonance cholangiopancreatography (MRCP), where upfront RFA can target intraductal components and reduce the risk of incomplete ablation or ductal injury.

### Procedural details

The rationale for the sequential RFA-first strategy is threefold. First, RFA reduces tumor size and vascularity, which may decrease the risk of post-EP bleeding and incomplete resection, particularly for tumors >3 cm. Second, for lesions with intraductal extension or peri-diverticular location, upfront RFA modifies the tumor configuration, making subsequent EP technically easier and safer. Third, the interval between RFA and EP (≥1 month) allows for pathological reassessment and inflammation resolution, potentially reducing postprocedural pancreatitis risk by avoiding simultaneous extensive manipulation of the papilla.

All patients underwent thorough preprocedural evaluations to exclude lesions unsuitable for endoscopic treatment and adjust the surgical plan. EP was deferred if lesions showed malignant features (rigidity, friability, easy bleeding, or ulceration), regardless of prior benign biopsy results, as biopsies can underestimate malignancy (6, 23, 24). The size of duodenal papillary adenomas is typically evaluated using endoscopic ultrasonography (EUS), which accurately measures tumor dimensions and intraductal extension. On EUS, papillary adenomas appear as well-defined hypoechoic or isoechoic lesions in the ampullary region. High-frequency EUS aids in assessing ductal wall involvement and identifying malignant features, such as irregular hypoechoic areas or tissue infiltration. Magnetic resonance cholangiopancreatography (MRCP) was performed in most cases to assess bile and pancreatic ducts, including the feasibility of prophylactic pancreatic stent placement. Contrast-enhanced Computed Tomography (CT) was utilized for larger tumors to assess possible lymph node metastasis.

All procedures were performed by two senior endoscopists. Patients fasted for at least 12 h before the procedure, which was conducted under sedation or general anesthesia with anesthesiologist monitoring. EP consisted of four steps (Supplementary Video S1): (1) Submucosal injection. A small-volume injection of methylene blue-stained saline (<1 mL per site) was administered. Although some studies suggest that submucosal injection may lower en bloc resection rates (17, 25, 26). Whereas recent reports indicate that targeted injection reduces bleeding without compromising resection (27, 28). Injection was halted upon slight lesion elevation to prevent the “buried effect.” (2) Snare resection. Lesions were resected using “ENDOCUT” mode (Q effect: 3, cut duration: 1, cut interval: 6) (ERBE, Tübingen, Germany). (3) Duct cannulation and stenting. After resection, bile and pancreatic duct cannulation was attempted. Contrast confirmed the absence of intraductal involvement. Pancreatography was generally avoided to reduce postprocedural pancreatitis risk. Stents (usually 5 Fr-5 cm single-pigtail stents, COOK Medical, USA) were placed based on successful cannulation. (4) Closure of the resection plane. Titanium clips were applied. If a pancreatic stent was placed, the first clip was positioned near the stent. If only biliary stenting was achieved, clips were positioned to avoid the pancreatic orifice.

The RFA procedure (Supplementary Video S2) involved three steps: (1) Duct cannulation and dual guidewire placement. (2) RFA using the Habib EndoHPB catheter (Boston Scientific) at 7 W in soft coagulation mode for 30–90 s, applied until two-thirds of the lesion turned white without extending beyond the ampullary base to avoid perforation or strictures. (3) Post-RFA stenting. Dual stenting of the bile and pancreatic ducts was performed, typically using a 5Fr-5 or 5Fr-3 cm pancreatic stent and an 8.5Fr-7 cm or 5Fr-5 cm biliary stent.

All patients were hospitalized, with postoperative monitoring for pancreatitis, perforation, and bleeding, followed by appropriate management as required.

### Surveillance protocol

All patients followed a structured surveillance protocol. The first endoscopy was performed approximately 2 months post-procedure to assess residual lesions. Subsequent duodenoscopy with biopsy was scheduled every 6 months to monitor for recurrence. Follow-up continued for at least 5 years. For patients with biliary or pancreatic duct stents, an abdominal plain radiograph was taken within the first month to check for spontaneous stent dislodgement. If the stent had not spontaneously dislodged, it was endoscopically removed. If residual or recurrent lesions were detected, RFA was scheduled.

### Statistical analysis

Frequencies and percentages were calculated for categorical variables, such as prophylactic pancreatic duct (PDT) stenting, prophylactic bile duct (BDT) stenting, and initial treatment modality. Mean ± Interquartile Range (IQR) was calculated for continuous variables, such as the number of EP and RFA sessions and the total number of treatment sessions. Binary logistic regression was conducted to identify risk factors for pancreatitis, intraprocedural bleeding, and postprocedural bleeding. Patients were divided into RFA and non-RFA group to evaluate RFA safety. Owing to the small number of RFA cases, propensity score matching was used to reduce confounding. Subsequently, analysis of variance (ANOVA) was then performed to assess differences in adverse events incidence between the two groups. All analyses were performed using SPSS (version 27.0, IBM Corp., Armonk, NY, USA).

## Results

### Patient demographics and clinical characteristics

This study included 132 patients (61 females [46.2%], 71 males [53.8%]) with a mean age of 52.2 ± 11.8 years (range, 27–75). Demographic and clinical characteristics are summarized in Supplementary Table S1. Alcohol consumption was reported by 20 patients (15.2%) and smoking by 17 (12.9%). Only two patients (1.5%) were on oral anticoagulants. Most lesions were sporadic (131, 99.2%), with one patient (0.8%) having familial adenomatous polyposis (FAP). Six patients (4.5%) reported abdominal pain, and none had jaundice. Comorbidities included hypertension (21 patients, 15.9%), diabetes mellitus (9, 6.8%), and cardiovascular disease (3, 2.3%).

### Tumor characteristics

The mean tumor size was 1.62 ± 0.57 cm (range 0.7–4.0 cm) (Supplementary Table S2). Most tumors were adenomas (117 patients, 88.6%), with tubular adenomas in 101 patients (76.5%) and tubulovillous adenomas in 16 (12.1%). One patient (0.8%) had a neuroendocrine tumor (NET), and 14 (10.6%) had chronic inflammation. Two patients (1.5%) had adenocarcinoma, four (3.0%) with high-grade dysplasia, 104 (78.8%) with low-grade dysplasia, and 21 (15.9%) had no dysplasia.

### Treatment modalities and outcomes

**Figure 1:**
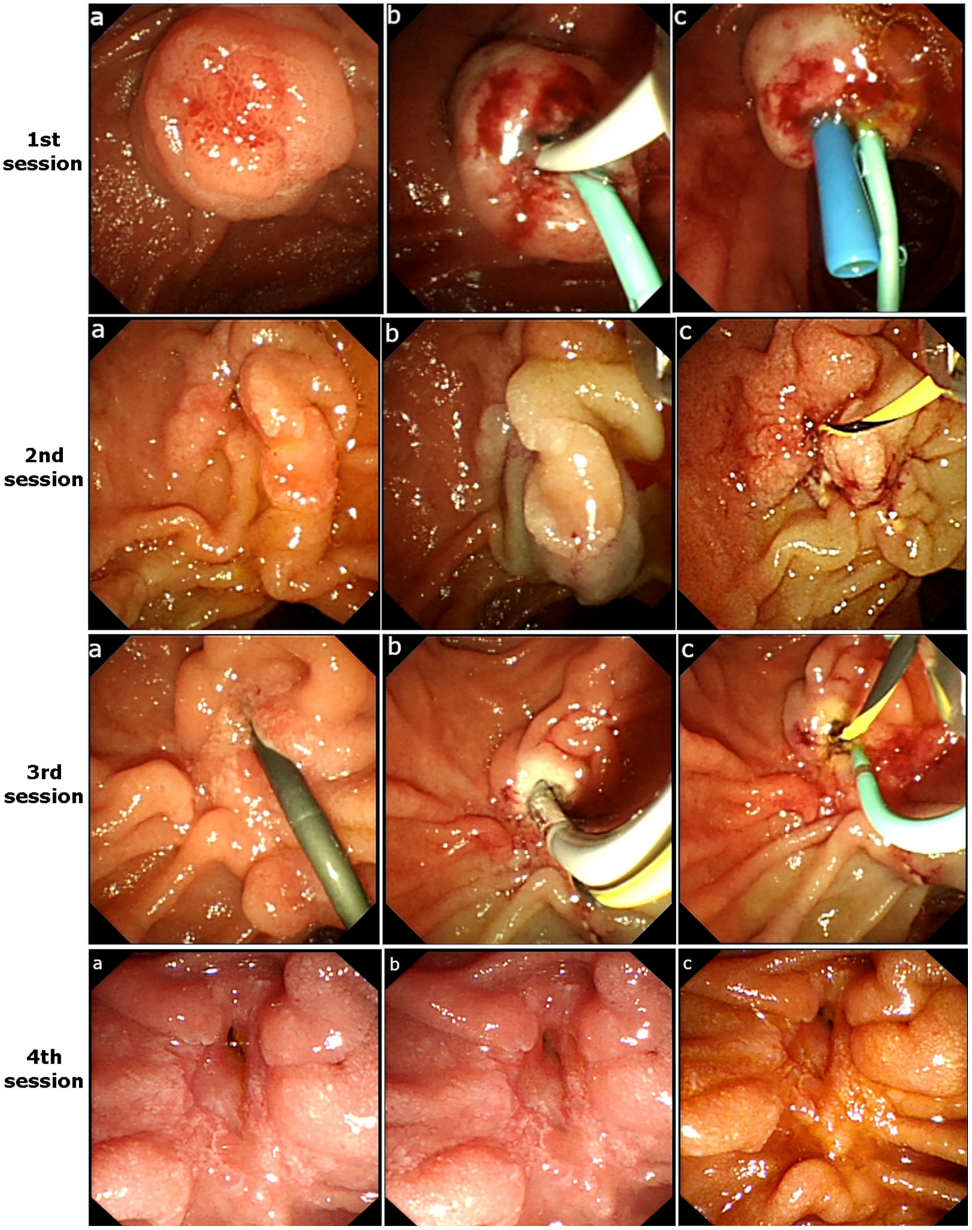
Four treatment sessions for a patient who received RFA as the initial treatment due to the tumor >3 cm. 1st session: **(a)** Ampullary tumor before treatment; **(b)** After placing a pancreatic duct stent, RFA was performed under the guidance of a bile duct wire; **(c)** Post-RFA placement of bile and pancreatic duct stents. 2nd session: **(a)** Significant reduction in ampullary tumor size before the second treatment, with residual tumor tissue observed on the anal side of the bile and pancreatic duct orifice; **(b)** Disc-shaped tumor tissue observed after submucosal injection; **(c)** Post-EP placement of a pancreatic duct guidewire in preparation for pancreatic duct stent placement. 3rd session: **(a)** Retained pancreatic duct stent with residual tumor tissue adjacent to the stent prior to the third treatment; **(b)** After biopsy, the pancreatic duct stent was removed and double guidewires were placed in the bile and pancreatic ducts, followed by RFA via the bile duct guidewire; **(c)** Placement of a pancreatic duct stent, with plans to insert a bile duct plastic stent via the guidewire. 4th session: **(a)** Follow-up endoscopy showing complete disappearance of the original tumor with scarring and visualization of the bile and pancreatic duct orifice; **(b)** Scar tissue at the site of the original ampullary tumor was biopsied; **(c)** Smooth bile flow observed after endoscopic suction.

Table 1 summarize treatment modalities and outcomes. Prophylactic pancreatic duct stenting was performed in 108 patients (81.8%), and prophylactic bile duct stenting in 88 patients (66.7%). The initial treatment was EP in 123 patients (93.2%), with curative resection in 117. Remnant lesions were identified in four patients; two underwent PD, and two were treated with RFA (one case is presented in Supplementary Figure S2). Recurrent lesions were detected in two patients, treated with PD and RFA, respectively. RFA was the initial treatment in nine patients (6.8%), including two patients with tumors >3 cm (Figure 1), three with peri-diverticular lesions, four with intraductal invasive lesions. One patient with an intraductal lesion underwent PD after post-RFA biopsy revealed adenocarcinoma. 129 patients (97.7%) were cured after one EP session, and one patient (0.8%) required two sessions. For RFA, one session was sufficient in 7 patients (5.3%), two sessions were required in 3 (2.3%), and three sessions in 2 (1.5%). Technical success was achieved in all patients (100%). The overall short-term clinical success rate was 96.6% (114/118), 4 patients who underwent PD were considered clinical failures. Fourteen patients with chronic inflammation were excluded from the short-term clinical success analysis, leaving 118 in the final cohort.

**Table 1:** Treatment modalities and outcomes.

Variables	*n* (%) or *n*
Duct stenting	
Prophylactic PDT stenting *n* (%)	108 (81.8%)
Prophylactic BDT stenting *n* (%)	88 (66.7%)
Initial treatment	
EP *n* (%)	123 (93.2%)
Curative resected *n*	117
Remnant lesions *n*	4
Pancreatoduodenectomy *n*	2
RFA *n*	2
Recurrent lesions *n* (%)	2
Pancreatoduodenectomy *n*	1
RFA *n*	1
RFA *n* (%)	9 (6.8%)
Tumor size >3 cm *n*	2
Peri-diverticular lesions *n*	3
Intraductal lesions *n*	4
Pancreatoduodenectomy *n*	1
EP sessions *n* (%)	130 (98.5%)
1 session *n* (%)	129 (97.7%)
2 sessions *n* (%)	1 (0.8%)
RFA sessions *n* (%)	12 (9.1%)
1 session *n* (%)	7 (5.3%)
2 sessions *n* (%)	3 (2.3%)
3 sessions *n* (%)	2 (1.5%)
Technical success	132 (100%)
*Short-term clinical success	114 (96.6%)

*When calculating the proportion of short-term clinical success, 14 patients with histological findings of chronic inflammation were excluded from the total count, resulting in a final cohort of 118 patients.

### Adverse events and follow-up

A total of 27 patients (20.5%) experienced adverse events (Table 2), with postprocedural pancreatitis being the most common, affecting 17 patients (12.9%). Except for one with moderate pancreatitis, most had mild pancreatitis. All responded well to conservative management. Intraprocedural bleeding was observed in 8 patients (6.1%) and was effectively managed with endoscopic hemostasis. Postprocedural bleeding occurred in seven patients (5.3%), all treated successfully with repeat endoscopic hemostasis. One perforation case (0.8%) was resolved with endoscopic clip placement and conservative care, leading to discharge (Supplementary Video S3). No cases of stenosis were observed during the short-term follow-up period (mean 7.9 months). The mean follow-up was 7.9 ± 5.53 months (range 2–32 months). During short-term follow-up, remnant lesions were found in 7 patients (5.9%) and recurrent lesions in 2 patients (1.7%).

**Table 2:** Adverse events and follow-up.

Variable	*n* (%)
Adverse events *n* (%)	27 (20.5%)
Intraprocedural bleeding *n* (%)	8 (6.1%)
Postprocedural bleeding *n* (%)	7 (5.3%)
Postprocedural pancreatitis *n* (%)	17 (12.9%)
Perforation *n* (%)	1 (0.8%)
Stenosis *n* (%)	0 (0%)
Follow-up (month)	
Mean ± SD	7.9 ± 5.53
Range	2–32
Median (IQR)	6.2 (4.1–10.3)
*Remnant lesions *n* (%)	7 (5.9%)
*Recurrent lesions *n* (%)	2 (1.7%)

*When calculating the proportion of residual and recurrent lesions, 14 patients with histological findings of chronic inflammation were excluded from the total count, resulting in a final cohort of 118 patients.

### Logistic regression analysis of risk factors associated with adverse events

Binary logistic regression was conducted to identify risk factors for pancreatitis, intraprocedural bleeding, and delayed bleeding. Owing to the limited cases, analysis for perforation and stenosis was not feasible. The risk factors associated with pancreatitis were presented in Supplementary Table S3. Prophylactic BDT stenting reduced pancreatitis risk (OR 0.63, 95% CI: 0.05–7.39, *p* = 0.02), and prophylactic PDT stenting demonstrated a statistically significant protective effect (OR 0.000, 95% CI: 0.000, *p* < 0.001). Larger tumor size increased the risk (OR 2.01, 95% CI: 0.48–8.4, *p* = 0.03). No other factors were significantly linked to pancreatitis risk. The analysis of risk factors of intraprocedural bleeding revealed tumor size as the only significant factor (OR 9.321, 95% CI: 1.995–43.553, *p* < 0.001), highlighting higher bleeding risk with larger tumors (Supplementary Table S4). Other factors, such as prophylactic BDT stenting and PDT stenting were not significantly associated factors. The analysis for postprocedural bleeding shown prophylactic BDT stenting significantly reduced bleeding risk (OR 0.045, 95% CI, 0.003–0.652; *p* = 0.028), as did prophylactic PDT stenting (OR 0.199, 95% CI, 0.017–2.291; *p* = 0.006). Tumor size (OR 6.334, 95% CI: 0.873–45.940, *p* = 0.024) was also a significant risk factor (Supplementary Table S5).

### Patient demographics and clinical characteristics for RFA group

The efficacy and safety of the RFA technique were assessed by comparing 12 patients (9.1%) in the RFA group with 120 patients (90.9%) in the EP-only group. In the RFA group, 33.3% were female (*n* = 4) and 66.7% male (*n* = 8), with a mean age of 56.2 ± 3.10 years (range 34–72 years). Mean tumor size was 2.3 ± 0.10 cm (range 1.9–3.2 cm). All patients (*n* = 12) had adenomas (33.3% tubular adenoma, 66.7% tubulovillous adenoma) and received prophylactic PDT and BDT stenting. Most RFA patients (*n* = 9, 75%) required two treatment sessions, while 25% (*n* = 3) needed four. For RFA sessions, 58.3% (*n* = 7) had one, 25% (*n* = 3) had two, and 16.7% (*n* = 2) had three. Adverse events were observed in 16.7% (*n* = 2), with intraprocedural bleeding, postprocedural bleeding, and postprocedural pancreatitis each occurring in 8.3% (*n* = 1). To date, no cases of stenosis have been reported. Technical success was achieved in all patients (*n* = 12, 100%), and short-term clinical success was observed in 91.7% (*n* = 11). One patient with an intraductal lesion underwent PD after post-RFA biopsy, which revealed an adenocarcinoma. The mean follow-up period was 10.9 ± 2.11 months (2–24 months) (Table 3).

**Table 3:** Characteristics in the RFA group.

Characteristic	Value
Gender	
Female *n* (%)	4 (33.3%)
Male *n* (%)	8 (66.7%)
Age (years)	
Mean ± SD	56.2 ± 3.10
Range	34–72
Tumor size (cm)	
Mean ± SD	2.3 ± 0.10
Range	1.9–3.2
Histology type	
Adenoma *n* (%)	12 (100%)
Tubular adenoma *n* (%)	4 (33.3%)
Tubulovillous adenoma *n* (%)	8 (66.7%)
Prophylactic PDT stenting *n* (%)	12 (100%)
Prophylactic BDT stenting *n* (%)	12 (100%)
Total treatment sessions	
2 session *n* (%)	9 (75%)
4 sessions *n* (%)	3 (25%)
RFA sessions	
1 session *n* (%)	7 (58.3%)
2 sessions *n* (%)	3 (25%)
3 sessions *n* (%)	2 (16.7%)
Adverse events, *n* (%)	2 (16.7%)
Intraprocedural bleeding, *n* (%)	1 (8.3%)
Postprocedural bleeding, *n* (%)	1 (8.3%)
Postprocedural pancreatitis, *n* (%)	1 (8.3%)
Stenosis, *n* (%)	0 (0%)
Technical success	12 (100%)
Short-term clinical success	11 (91.7%)
Follow-up (month)	
Mean ± SD	10.9 ± 2.11
Range	2–24

### Comparison of efficacy and safety between RFA and EP treatment

Owing to the small number of cases using RFA, which resulted in a significant disparity in the number of cases between the two groups. Propensity score matching was employed to control for confounding factors including tumor size, age, and sex, resulting in nine EP-only patients matched to the RFA group, forming a new non-RFA group. Baseline characteristics showed no significant differences in tumor size, age or gender composition between groups and after matching (*p* > 0.05). After confirming the balance between the groups, ANOVA was then performed to compare the incidence of adverse events between the RFA and non-RFA groups (Supplementary Figure S3). Statistical analysis showed that the comparison of adverse events between RFA group 16.7% (*n* = 2) and non-RFA group 1.1% (*n* = 1) showed no significant difference (*p* = 0.719) (Table 4). Intraprocedural bleeding was observed in 8.3% (*n* = 1) of the RFA group and 11.1% (*n* = 1) of the non-RFA group (*p* = 0.830). Postprocedural bleeding and pancreatitis were observed in 8.3% (*n* = 1) of the RFA group, with no cases in the non-RFA group (*p* = 0.375). The observed difference in adverse events between the RFA and non-RFA groups was not statistically significant, but this finding should be interpreted with caution due to limited statistical power and does not confirm the safety of RFA treatment for ampullary tumors. Moreover, given the limited number of patients treated with upfront RFA (*n* = 12) and the retrospective design, this investigation is best viewed as a hypothesis-generating, proof-of-concept study aimed at exploring the feasibility and safety of a sequential RFA-first strategy for complex ampullary tumors. Direct comparisons between the RFA and EP groups, despite propensity score matching, should be interpreted with caution due to unavoidable confounding by indication.

**Table 4:** Comparing the adverse events between RFA and Non-RFA groups.

Outcome	RFA group	Non-RFA group	*p*-value
Adverse events, *n* (%)	2 (16.7%)	1 (11.1%)	0.719
Intraprocedural bleeding, *n* (%)	1 (8.3%)	1 (11.1%)	0.830
Postprocedural bleeding, *n* (%)	1 (8.3%)	0 (0%)	0.375
Postprocedural pancreatitis, *n* (%)	1 (8.3%)	0 (0%)	0.375

## Discussion

EP is a minimally invasive treatment option for ampullary tumors. With continuous advancements in endoscopy technology, the success rate of EP has gradually improved, with a long-term cure rate reaching up to 80% (3). Moreover, postoperative complications can be solved by conservative treatment or endoscopic intervention (29). Consequently, EP has emerged as a primary treatment option for ampullary tumors. Nonetheless, due to the unique anatomical location of the duodenal papilla and the technical complexity of the procedure, a standardized endoscopic approach has yet to be established. Key considerations include the prevention and management of bleeding and perforation; the use of pancreatic duct and bile duct stents to prevent acute pancreatitis, and the application of RFA for larger tumors prior to EP. In this retrospective study, we systematically analyzed the medical records, clinical features, treatment methods, and complications of ampullary tumors, and compared the safety and effectiveness of EP and RFA in treating ampullary tumors. This sequential strategy of upfront RFA followed by EP provides a safe and feasible alternative for patients who may otherwise require surgical intervention. This is especially valuable in complex scenarios such as peri-diverticular tumors, recurrent lesions, and ampullary tumors with intraductal extension. Importantly, given the retrospective design and small sample size of the RFA group, our findings should be interpreted as preliminary evidence of feasibility and safety, rather than definitive comparative effectiveness between EP and RFA.

The high short-term clinical success rates (91.7 and 96.6%) and low complication rates (16.7 and 20.5%) in both the RFA subgroup and the overall cohort underscore the potential of this approach to extend the indications for endoscopic treatment, particularly when EP alone is technically demanding or less effective. To our knowledge, the use of RFA as an exclusive initial treatment prior to EP, rather than as an adjunct for residual lesions, has been rarely reported. Our study provides preliminary evidence supporting this sequential approach. Previous studies have primarily investigated RFA after EP or in recurrent cases (8, 10–12, 16, 18, 20, 22). In contrast, our novel strategy employs RFA at the outset to reduce procedural complexity and improve outcomes. Moreover, the complication rates between RFA and non-RFA groups were comparable, with overall adverse event rates showing no significant difference (16.7% vs. 11.1%, *p* = 0.719). These findings further support that preliminary RFA or staged supplementation of RFA may represent an effective and safe approach to minimizing adverse events during ampulla tumor management.

This study achieved a higher short-term clinical success rate (96.6%) compared with the previously reported range of 70–95% (2, 6, 23), which can be attributed to the broader definition of success adopted in our protocol. Unlike prior investigations that relied on a single treatment session, we allowed multiple treatment sessions, defining success as endoscopic manageability over repeated interventions. This multisession approach is pivotal for ampullary tumors, as complex cases may necessitate multiple procedures to achieve complete resection and long-term disease control.

Our study also demonstrated a lower adverse events rate (20.5%) than the reported 25–35% (21, 30), likely reflecting differences in patient selection, tumor profiles, and prophylactic measures. Notably, postprocedural bleeding was 2.3%, substantially lower than the 10 to 24% reported elsewhere (4, 12, 15). This outcome is largely attributable to our routine use of prophylactic clip closure following EP. Similar findings by Park et al. (15) and Choi et al. (4) reinforce the protective role of prophylactic clipping against bleeding. Although Kagawa highlighted the technical difficulties of using clips with a duodenoscope, we successfully achieved closure in our cohort. Furthermore, prophylactic BDT and PDT stenting significantly reduced postprocedural bleeding, by facilitating more effective clip closure of the resection plane after stent placement. In cases where stent placement was unsuccessful, partial clipping left the resection plane incompletely sealed, thereby increasing bleeding risk. Postprocedural pancreatitis occurred in 12.9% of our patients, aligning with the 5–15% range described in previous reports (12, 15, 30), while the perforation rate was 2.3%, consistent with the 0.5–2% range (12, 15, 31). No biliary strictures were observed in the RFA group, a finding that contrasts with the reported 5–15% incidence of strictures (8, 10, 12, 19, 20, 32–34). This discrepancy could be explained by the use of low-power settings (Erbe 200D, 7 W, soft coagulation) and tightly controlled RFA duration (30–90 s), with ablation halted once more than half (or up to two-thirds) of the tumor turned white to minimize injury to the bile duct wall. Additionally, the small sample size (12 cases) and relatively short follow-up period (10.9 ± 2.11 months) may not have captured late-onset strictures, thus influencing our observations. Therefore, conclusions regarding stricture risk should be limited to the early postprocedural period, and long-term safety remains to be determined.

Additionally, our data suggest that tumor size is a critical risk factor for complications during the treatment of ampullary adenomas. Larger tumors are more challenging to resect endoscopically and are therefore associated with higher rates of bleeding, perforation, and other adverse events. By applying RFA before EP, the tumor volume can be reduced, potentially lowering procedural complexity and minimizing complication rates. These findings are consistent with other reports indicating that tumor size influences treatment-related morbidity (4, 21). Consequently, our results reinforce the notion that preliminary use of RFA or staged RFA supplementation may be both effective and safe in reducing adverse events for patients with large ampullary tumors, further supporting its adoption as a viable treatment strategy in selected cases.

In conclusion, our study suggests that sequential use of EP and RFA may represent a potentially safe and effective approach for managing ampullary tumors in select patients with complex features (e.g., large size, peri-diverticular location, or intraductal extension). However, given the study’s inherent limitations, these conclusions are preliminary and require confirmation in larger, prospective cohorts. Nonetheless, several limitations should be noted. First, the retrospective, single-center design and the substantial sample size imbalance between the EP and RFA groups (123 vs. 12) introduce potential selection bias and confounding by indication. Patients receiving RFA as initial treatment presented with more complex tumor characteristics (e.g., size >3 cm, peri-diverticular location, or intraductal extension), which inherently limits direct comparability between the two groups. Second, the small sample size in the RFA subgroup (*n* = 12) reduced the statistical power to detect minor differences between patient groups, and unmeasured confounders (e.g., operator experience, detailed anatomical variations) may persist despite propensity score matching. Finally, the absence of long-term follow-up data precludes definitive conclusions about the durability of these treatment outcomes. Therefore, our findings should be interpreted as preliminary evidence supporting the feasibility and safety of sequential RFA and EP as an alternative strategy for high-risk ampullary tumors, rather than a robust comparative effectiveness study. Future multicenter, prospective investigations with larger sample sizes and extended follow-up are warranted to validate and refine these findings.

## Data Availability

The original contributions presented in the study are included in the article/Supplementary material, further inquiries can be directed to the corresponding author.

## Supplementary material

SUPPLEMENTARY FIGURE S1 The Flowchart of this research. RFA, radiofrequency ablation; EP, endoscopic papillectomy; PD, pancreatoduodenectomy.

SUPPLEMENTARY FIGURE S2 Five treatment sessions for a patient with a remnant lesion following EP. 1st session: (a) Ampullary tumor before treatment; (b) During EP; (c) Residual tumor tissue at the orifice of the bile and pancreatic ducts after EP (residual tumor tissue was removed with biopsy forceps, video 3). 2nd session: (a) Residual lesion and retained bile and pancreatic duct stents observed prior to the second treatment; (b) RFA performed after the removal of the bile and pancreatic duct stents; (c) Placement of bile and pancreatic duct stents post-procedure. 3rd session: (a) Residual tumor tissue observed prior to the third treatment; (b) RFA performed after biopsy; (c) Post-procedure view of the treatment site (with subsequent placement of bile and pancreatic duct stents). 4th session: (a) Irregular ampullary mucosa prior to the fourth treatment; (b) RFA performed after biopsy; (c) Post-RFA placement of double guidewires in preparation for bile and pancreatic duct stents. 5th session: (a) Visualization of the minor papilla (outlined by the green rectangular dashed line) and the major papilla (outlined by the red elliptical solid line); (b) Only the orifice of the bile and pancreatic ducts at the major papilla (outlined by the red elliptical solid line) and the minor papilla (outlined by the green rectangular dashed line) are observed; (c) Visualization of the bile duct orifice at the major papilla (outlined by the red elliptical solid line).

SUPPLEMENTARY FIGURE S3 Flowchart of patient categorization or study population. RFA, radiofrequency ablation.
