# Supplementary figures and images for: A sequential strategy of upfront radiofrequency ablation followed by endoscopic papillectomy for complex ampullary tumors
Source: Front Med (Lausanne). 2026 Jun 19;13:1835891. doi: 10.3389/fmed.2026.1835891 (PMC13328028; doi:10.3389/fmed.2026.1835891)

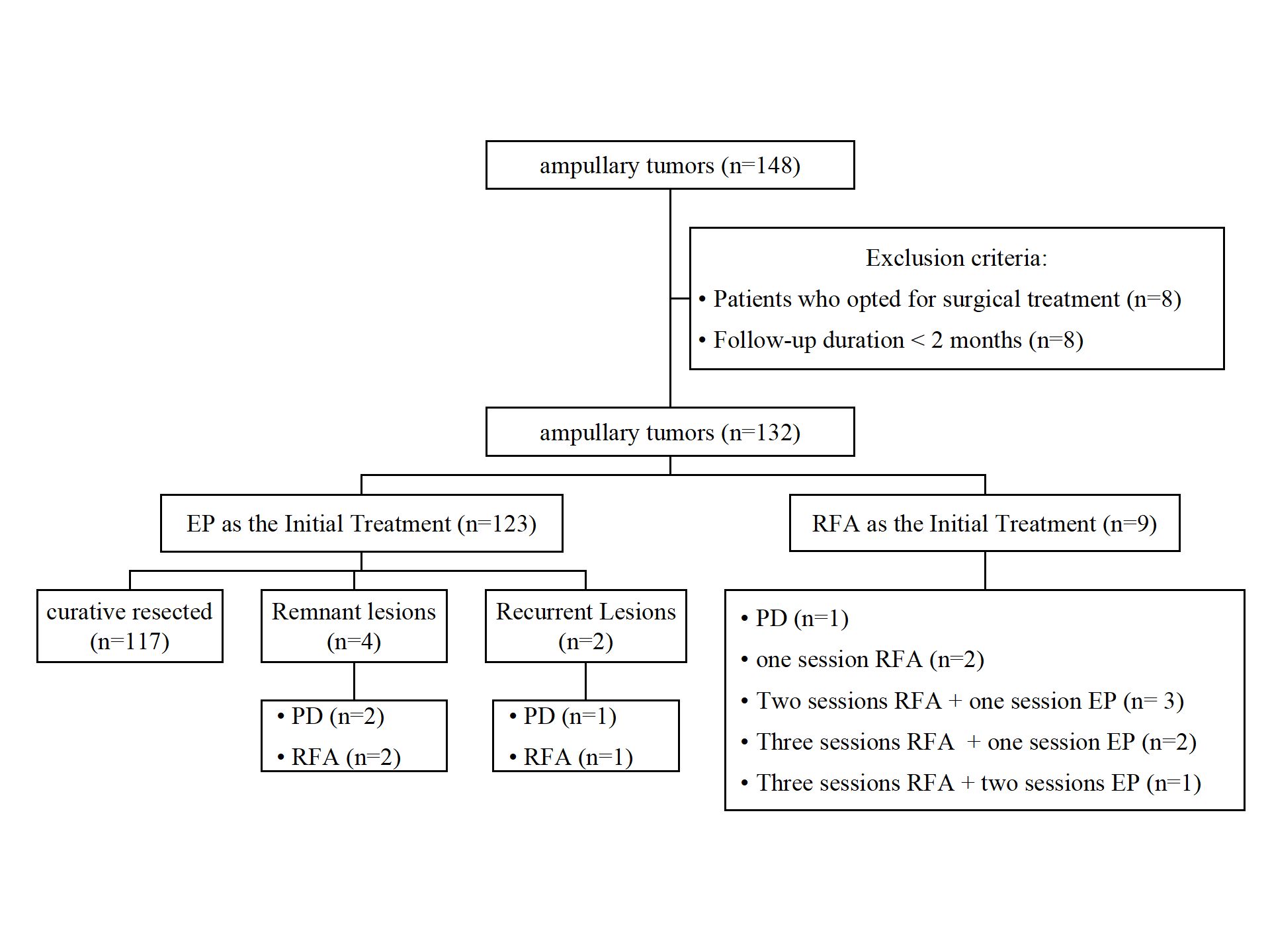

Supplement: SUPPLEMENTARY FIGURE S1 — The Flowchart of this research. RFA, radiofrequency ablation; EP, endoscopic papillectomy; PD, pancreatoduodenectomy. [file Image_1.TIF]

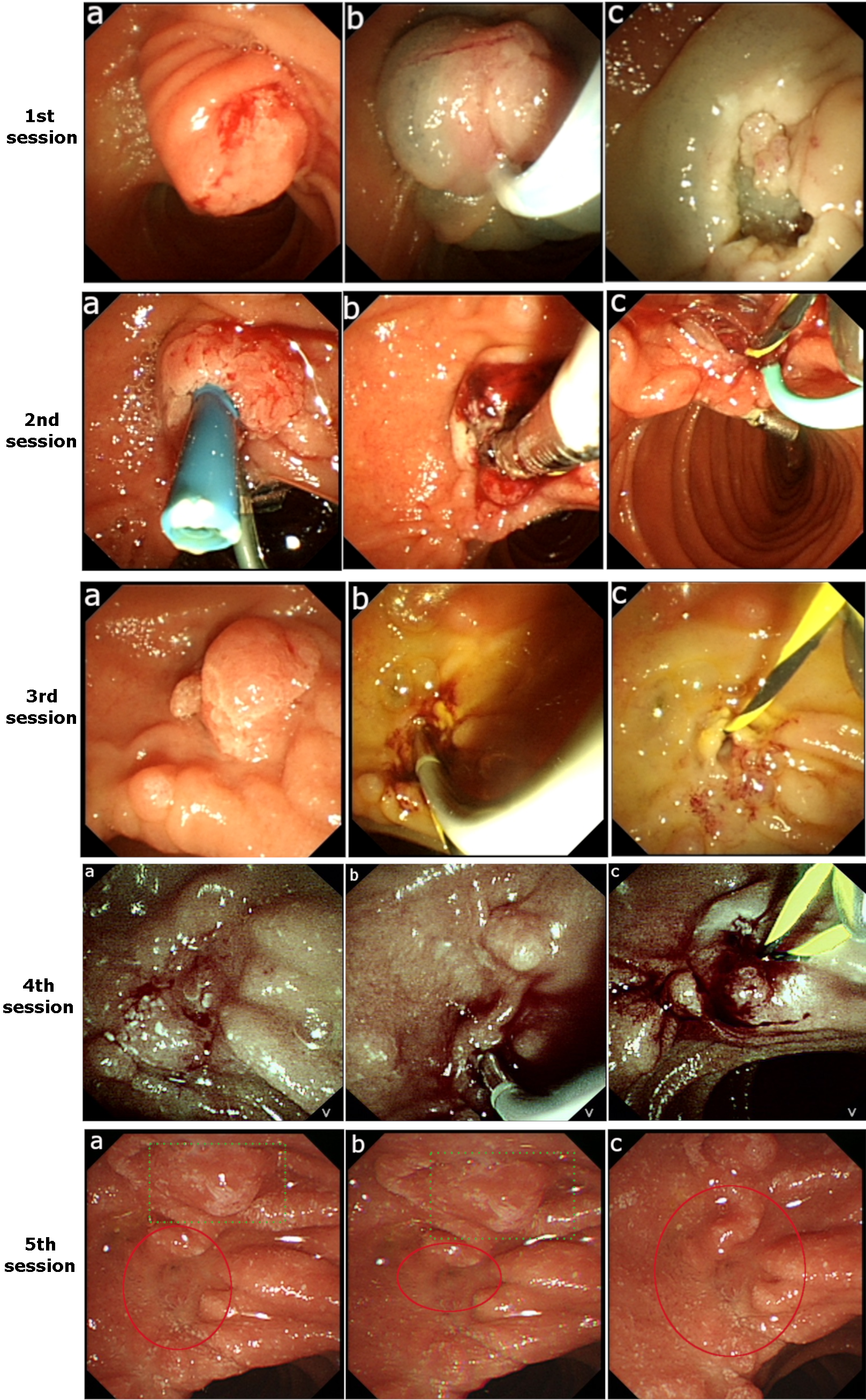

Supplement: SUPPLEMENTARY FIGURE S2 — Five treatment sessions for a patient with a remnant lesion following EP. 1st session: (a) Ampullary tumor before treatment; (b) During EP; (c) Residual tumor tissue at the orifice of the bile and pancreatic ducts after EP (residual tumor tissue was removed with biopsy forceps, video 3). 2nd session: (a) Residual lesion and retained bile and pancreatic duct stents observed prior to the second treatment; (b) RFA performed after the removal of the bile and pancreatic duct stents; (c) Placement of bile and pancreatic duct stents post-procedure. 3rd session: (a) Residual tumor tissue observed prior to the third treatment; (b) RFA performed after biopsy; (c) Post-procedure view of the treatment site (with subsequent placement of bile and pancreatic duct stents). 4th session: (a) Irregular ampullary mucosa prior to the fourth treatment; (b) RFA performed after biopsy; (c) Post-RFA placement of double guidewires in preparation for bile and pancreatic duct stents. 5th session: (a) Visualization of the minor papilla (outlined by the green rectangular dashed line) and the major papilla (outlined by the red elliptical solid line); (b) Only the orifice of the bile and pancreatic ducts at the major papilla (outlined by the red elliptical solid line) and the minor papilla (outlined by the green rectangular dashed line) are observed; (c) Visualization of the bile duct orifice at the major papilla (outlined by the red elliptical solid line). [file Image_2.TIF]

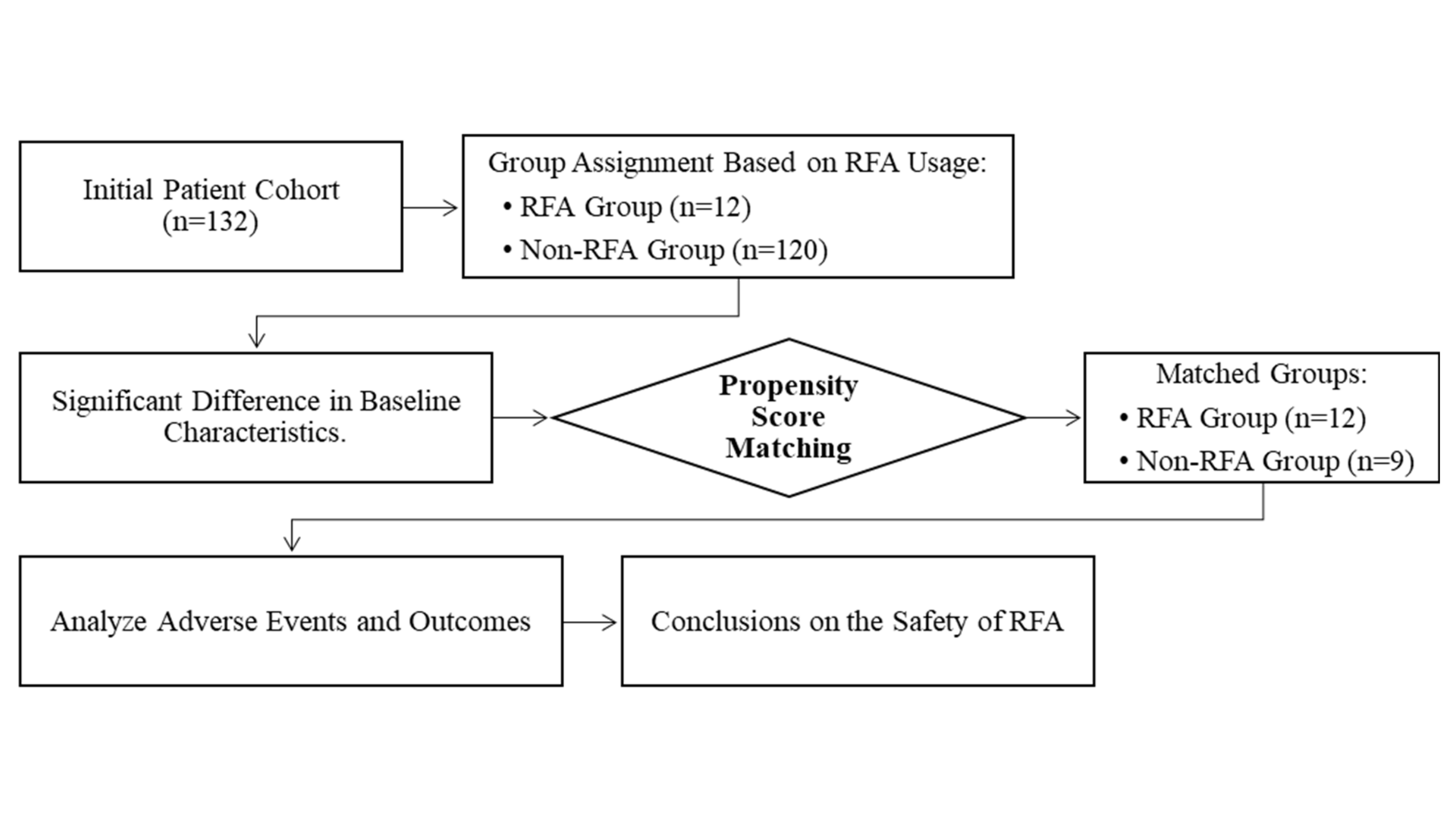

Supplement: SUPPLEMENTARY FIGURE S3 — Flowchart of patient categorization or study population. RFA, radiofrequency ablation. [file Image_3.TIF]
