# Supplementary material for: A sequential strategy of upfront radiofrequency ablation followed by endoscopic papillectomy for complex ampullary tumors
Source: Front Med (Lausanne). 2026 Jun 19;13:1835891. doi: 10.3389/fmed.2026.1835891 (PMC13328028; doi:10.3389/fmed.2026.1835891)
Supplement: Supplementary file 4 [file Table_1.DOCX]

Table S1. Patient demographics and clinical characteristics.

| Characteristic | n% |
| --- | --- |
| Gender |  |
| -Female n (%) | 61 (46.2%) |
| -Male n (%) | 71 (53.8%) |
| Age (years) |  |
| -Mean ± SD | 52.2 ± 11.8 |
| -Range | 27–75 |
| History of drinking n (%) | 20 (15.2%) |
| History of smoking n (%) | 17 (12.9%) |
| Oral anticoagulants n (%) | 2 (1.5%) |
| Sporadic lesions n (%) | 131 (99.2%) |
| FAP n (%) | 1 (0.8%) |
| Abdominal pain n (%) | 6 (4.5%) |
| Jaundice n (%) | 0 (0%) |
| Hypertension n (%) | 21 (15.9%) |
| Diabetes mellitus n (%) | 9 (6.8%) |
| Cardiovascular disease n (%) | 3 (2.3%) |
