# Supplementary material for: A sequential strategy of upfront radiofrequency ablation followed by endoscopic papillectomy for complex ampullary tumors
Source: Front Med (Lausanne). 2026 Jun 19;13:1835891. doi: 10.3389/fmed.2026.1835891 (PMC13328028; doi:10.3389/fmed.2026.1835891)
Supplement: Supplementary file 5 [file Table_2.DOCX]

Table S2. Tumor characteristics.

| Tumor size (cm) |  |
| --- | --- |
| -Mean ± SD | 1.62 ± 0.57 |
| -Range | 0.7–4.0 |
| Histology Type |  |
| -Adenoma n (%) | 117 (88.6%) |
| -Tubular Adenoma n (%) | 101 (76.5%) |
| -Tubulovillous Adenoma n (%) | 16 (12.1%) |
| -Neuroendocrine Tumor (NET) n (%) | 1 (0.8%) |
| -Chronic Inflammation n (%) | 14 (10.6%) |
| Dysplasia |  |
| -Adenocarcinoma n (%) | 2 (1.5%) |
| -High-grade Dysplasia n (%) | 4 (3.0%) |
| -Low-grade Dysplasia n (%) | 104 (78.8%) |
| -No Dysplasia n (%) | 21 (15.9%) |
