# Supplementary material for: A sequential strategy of upfront radiofrequency ablation followed by endoscopic papillectomy for complex ampullary tumors
Source: Front Med (Lausanne). 2026 Jun 19;13:1835891. doi: 10.3389/fmed.2026.1835891 (PMC13328028; doi:10.3389/fmed.2026.1835891)
Supplement: Supplementary file 6 [file Table_3.DOCX]

Table S3. Logistic regression analysis of risk factors associated with post-pancreatitis.

| Variable | Odds Ratio (OR) | 95% Confidence Interval (CI) | *P-*value |
| --- | --- | --- | --- |
| Initial Treatment | 5.357E+19 | 0.000 | 0.39 |
| Prophylactic BDT stenting | 0.63 | 0.05–7.39 | **0.02** |
| Prophylactic PDT stenting | 0.000 | 0.000 | **< 0.001** |
| Tumor size | 2.01 | 0.48–8.4 | **0.03** |
| Cardiovascular disease | 2.698E+23 | 0.000 | 0.50 |
| Diabetes mellitus | 1.449E+13 | 0.000 | 0.06 |
| Hypertension | 0.000 | 0.000 | 0.62 |
| Abdominal pain | 9295E+10 | 0.000 | 0.34 |
| FAP | 1.605E+11 | 0.000 | 0.70 |
| Oral anticoagulants | 0.000 | 0.000 | 0.58 |
| History of smoking | 3.184E+19 | 0.000 | 0.53 |
| History of drinking | 0.000 | 0.000 | 0.25 |
| Age | 1.010 | 0.92–1.11 | 0.24 |
| Gender | 0.379 | 0.03–5.01 | 0.55 |
