# Supplementary material for: A sequential strategy of upfront radiofrequency ablation followed by endoscopic papillectomy for complex ampullary tumors
Source: Front Med (Lausanne). 2026 Jun 19;13:1835891. doi: 10.3389/fmed.2026.1835891 (PMC13328028; doi:10.3389/fmed.2026.1835891)
Supplement: Supplementary file 7 [file Table_4.DOCX]

Table S4. Logistic regression analysis of risk factors associated with intraprocedural bleeding.

| Variable | Odds Ratio (OR) | 95% Confidence Interval (CI) | *P*-value |
| --- | --- | --- | --- |
| Initial Treatment | 0.000 | 0.000 | 0.430 |
| Prophylactic BDT stenting | 2.730 | 0.211–35.256 | 0.606 |
| Prophylactic PDT stenting | 0.650 | 0.049–8.629 | 0.606 |
| Tumor size | 9.321 | 1.995–43.553 | < 0.001 |
| Cardiovascular disease | 0.000 | 0.000 | 0.656 |
| Diabetes mellitus | 0.000 | 0.000 | 0.430 |
| Hypertension | 0.831 | 0.059–11.714 | 0.786 |
| Abdominal pain | 0.000 | 0.000 | 0.524 |
| FAP | 0.000 | 0.000 | 0.799 |
| Oral anticoagulants | 59304929.17 | 0.000 | 0.717 |
| History of smoking | 2.141 | 0.197–23.321 | 0.291 |
| History of drinking | 1.194 | 0.122–11.686 | 0.423 |
| Age | 1.029 | 0.932–1.136 | 0.505 |
| Gender | 1.306 | 0.167–10.199 | 0.825 |
