# Supplementary material for: A sequential strategy of upfront radiofrequency ablation followed by endoscopic papillectomy for complex ampullary tumors
Source: Front Med (Lausanne). 2026 Jun 19;13:1835891. doi: 10.3389/fmed.2026.1835891 (PMC13328028; doi:10.3389/fmed.2026.1835891)
Supplement: Supplementary file 8 [file Table_5.DOCX]

Table S5. Logistic regression analysis of risk factors associated with postprocedural bleeding.

| Variable | Odds Ratio (OR) | 95% Confidence Interval (CI) | *P*-value |
| --- | --- | --- | --- |
| Initial Treatment | 0.000 | 0.000 | 0.462 |
| Prophylactic BDT stenting | 0.045 | 0.003–0.652 | 0.028 |
| Prophylactic PDT stenting | 0.199 | 0.017–2.291 | 0.006 |
| Tumor size | 6.334 | 0.873–45.940 | 0.024 |
| Cardiovascular disease | 2.132 | 0.000 | 0.678 |
| Diabetes mellitus | 36.896 | 0.018–77120.623 | 0.421 |
| Hypertension | 0.000 | 0.000 | 0.237 |
| Abdominal pain | 0.000 | 0.000 | 0.553 |
| FAP | 0.000 | 0.000 | 0.812 |
| Oral anticoagulants | 0.000 | 0.000 | 0.736 |
| History of smoking | 9.947 | 0.448–221.099 | 0.015 |
| History of drinking | 0.191 | 0.004–9.706 | 0.309 |
| Age | 0.919 | 0.817–1.033 | 0.708 |
| Gender | 0.037 | 0.001–1.000 | 0.336 |
